# A mixed-methods study characterizing experiences of medical oncologists’ use of gonadotropin-releasing hormone agonists for treatment of breast cancer

**DOI:** 10.1007/s10549-025-07734-2

**Published:** 2025-06-03

**Authors:** Kimberley T. Lee, Bihe Hu, Dinorah Martinez Tyson, Carley Geiss, Susan T. Vadaparampil, Heather S. L. Jim, Clement K. Gwede, Hatem H. Soliman, N. Lynn Henry, Dawn L. Hershman

**Affiliations:** 1 Department of Breast Oncology, Moffitt Cancer Center & Research Institute, Tampa, FL USA; 2 Department of Health Outcomes and Behavior, Moffitt Cancer Center & Research Institute, Tampa, FL USA; 3 Department of Community and Family Health, University of South Florida, Tampa, FL USA; 4 Participant Research, Interventions and Measurement (PRISM) Core, Moffitt Cancer Center & Research Institute, Tampa, FL USA; 5 Division of Hematology/Oncology, Department of Internal Medicine, University of Michigan Medical School, Ann Arbor, MI USA; 6 Department of Breast Oncology, Herbert Irving Comprehensive Cancer Center at Columbia University, New York, NY USA; 7 Division of Hematology and Oncology, University of Pennsylvania, Philadelphia, 3400 Civic Center Blvd, PA 19104 USA

**Keywords:** Pre-menopausal, Breast cancer, Ovarian function suppression, Gonadotropin-releasing hormone agonist

## Abstract

**Purpose:**

The use of ovarian function suppression (OFS) for the treatment of breast cancer in pre-menopausal women is low and little is known about medical oncologist’ attitudes toward current guidelines pertaining to the use of OFS. This purpose of this study was to explore breast medical oncologists’ perceptions and use of gonadotropin-releasing hormone agonists as OFS for treatment of early-stage breast cancer.

**Methods:**

A quantitative survey exploring experiences with OFS was distributed to medical oncologists across the USA using mailing lists available through the American Medical Association. Survey responses were characterized using descriptive statistics.

**Results:**

Oncologists in this study reported high likelihood of recommending OFS for pre-menopausal women at high risk for recurrence of hormone receptor-positive early-stage breast cancer. In addition to tumor size, nodal involvement, and 21-gene recurrence scores, administration of chemotherapy was a strong surrogate for risk of recurrence. Concerns about treatment toxicity and patient hesitancy were the top barriers to OFS utilization. Oncologists also reported low confidence in their ability to determine menopausal status in the setting of amenorrhea post-chemotherapy (9% reported feeling very confident with this task) and to monitor ovarian function while on OFS.

**Conclusion:**

Oncologists reported strong agreement with established guidelines for the use of OFS in the treatment of early-stage hormone receptor-positive breast cancer. However, our findings indicate a need for guidance regarding the determination of menopausal status in the setting of amenorrhea and monitoring of ovarian function.

**Supplementary Information:**

The online version contains supplementary material available at 10.1007/s10549-025-07734-2.

## Introduction

In 2024, an estimated 16% of new invasive breast cancers in the USA will be diagnosed in women under the age of 50 [1]. Of these 49,000 cases, approximately 75% will be hormone receptor positive (HR +) [1]. Pre-menopausal women with HR + breast cancer were traditionally treated with the endocrine therapy (ET) tamoxifen to reduce the risk of breast cancer recurrence. However, data from the SOFT and TEXT clinical trials demonstrated that ovarian function suppression (OFS) in combination with tamoxifen or aromatase inhibitors improves 8-year disease-free survival in pre-menopausal women with HR + breast cancer compared to tamoxifen only. [2] Medically, OFS can be achieved with the use of gonadotropin-releasing hormone agonist (GnRHa) injections or surgically with oophorectomy.

In 2016, the American Society of Clinical Oncology (ASCO) issued a guideline update recommending that “higher-risk patients should receive ovarian suppression in addition to adjuvant endocrine therapy […]” [3]. The strongest recommendation was for women with stage II or III breast cancers who would typically receive chemotherapy. Women with stage I or II breast cancers at higher risk of recurrence, for whom chemotherapy may be considered, could also be considered for OFS. The National Comprehensive Cancer Network Clinical Practice Guidelines in Oncology (NCCN Guidelines®) for invasive breast cancer recommend consideration of OFS for pre-menopausal patients at higher risk of recurrence (i.e., young age, high-grade tumor, lymph node involvement) [4]. In fact, it may be that with the use of OFS, for some women there may not be additional benefit to chemotherapy. This question is currently the subject of the ongoing phase III clinical trial, OFSET [5].

However, use of OFS remains low with one retrospective cohort study demonstrating a 25% initiation rate of OFS among eligible young women with breast cancer diagnosed between 2014 and 2015 [6]. Limited data are available regarding oncologist attitudes and decision-making patterns regarding OFS. To address this important gap, we conducted a mixed-methods study to investigate how medical oncologists use OFS (defined here as GnRH agonists) for pre-menopausal women with early-stage HR + breast cancer.

## Methods

### Participant sample and recruitment

Oncologists were included if they self-identified as an English-speaking medical oncologist providing care to a panel with at least 15% of breast cancer cases. All participants were provided with a $100 gift card as a token of thanks for their time. This study was reviewed by Advarra’s Institutional Review Board and deemed to be research exempt (MCC 22572). Participants were provided with an information sheet describing the project.

Recruitment occurred between November 2023 and May 2024. Four oncologists who participated in semi-structured interviews were recruited through the authors’ professional networks using purposive and convenience sampling techniques. Oncologists who completed the survey were recruited using the American Medical Association (AMA) Masterfile and direct mailings. The AMA Masterfile is a database of all licensed practicing physicians in the USA and is the only national database of licensed practicing physicians. To expand the sampling frame, oncologists were also identified via practice websites and contacted when contact information was publicly available.

### Survey development and content

We conducted semi-structured interviews with 4 community and academic medical oncologists to contextualize their experiences with recommending and prescribing OFS with the purpose of identifying constructs for the survey. Interviews were conducted by one study team member (KTL).

The survey domains included characteristics that informed perceived risk of recurrence, determination of menopausal status, monitoring of ovarian status during treatment with ET/OFS, attitudes toward current guidelines regarding OFS, and barriers to adopting routine use of OFS for high-risk pre-menopausal patients. The survey items also assessed demographic characteristics (e.g., race/ethnicity, gender) and practice information (e.g., practice setting, years in practice).

### Data analysis

#### Qualitative analysis

Interview transcripts were systematically reviewed to identify themes related to oncologists’ conceptualizations of need for OFS and factors determining whether or not OFS is recommended. These themes were used to inform the development of clinical vignettes and questions used in the survey.

#### Quantitative analysis

Demographic characteristics and survey responses were described using frequencies and percentages. Associations between practitioners’ years of experience and practice type with various clinical decision-making factors and management approaches were explored using two-way frequency tables and analyzed using two-tailed Fisher’s exact tests. A significance level of *α* = 0.05 was established as a priori. All statistical analyses were performed using SAS version 9.4 (Cary, NC, USA).

## Results

A total of 56 oncologists participated in the survey portion of the study and their sociodemographic and practice characteristics are presented in Table 1. Surveys were sent to 2,968 oncologists from which we received 75 responses, yielding a response rate of 2.5%. Of the initial respondents, 62 were eligible for the study and 56 completed the survey. Six of the eligible respondents were lost to follow-up prior to survey completion. The gender distribution was relatively balanced with females representing slightly more than half of the sample (54%). Most oncologists were Non-Hispanic White (61%) and 23% identified as Asian, 7% as Black, and 4% as Hispanic. The majority (71%) of respondents had > 10 years of oncology work experience and practiced in an academic setting (64%).

**Table 1:** Provider demographics: n = 56

Variable	n(%)
Gender	
Female	30 (54%)
Male	26 (46%)
Race/ethnicity*	
Non-Hispanic White	34 (62%)
Asian	13 (24%)
Non-Hispanic Black	4 (7%)
Hispanic	2 (4%)
Other	2 (4%)
Practice years*	
> 10 years	40 (73%)
0–10 years	15 (27%)
Practice type	
Academic	36 (64%)
Non-academic	20 (36%)

^*^n = 55 (1 missing)

### Preferences regarding OFS based on clinical characteristics

**Fig. 1:**
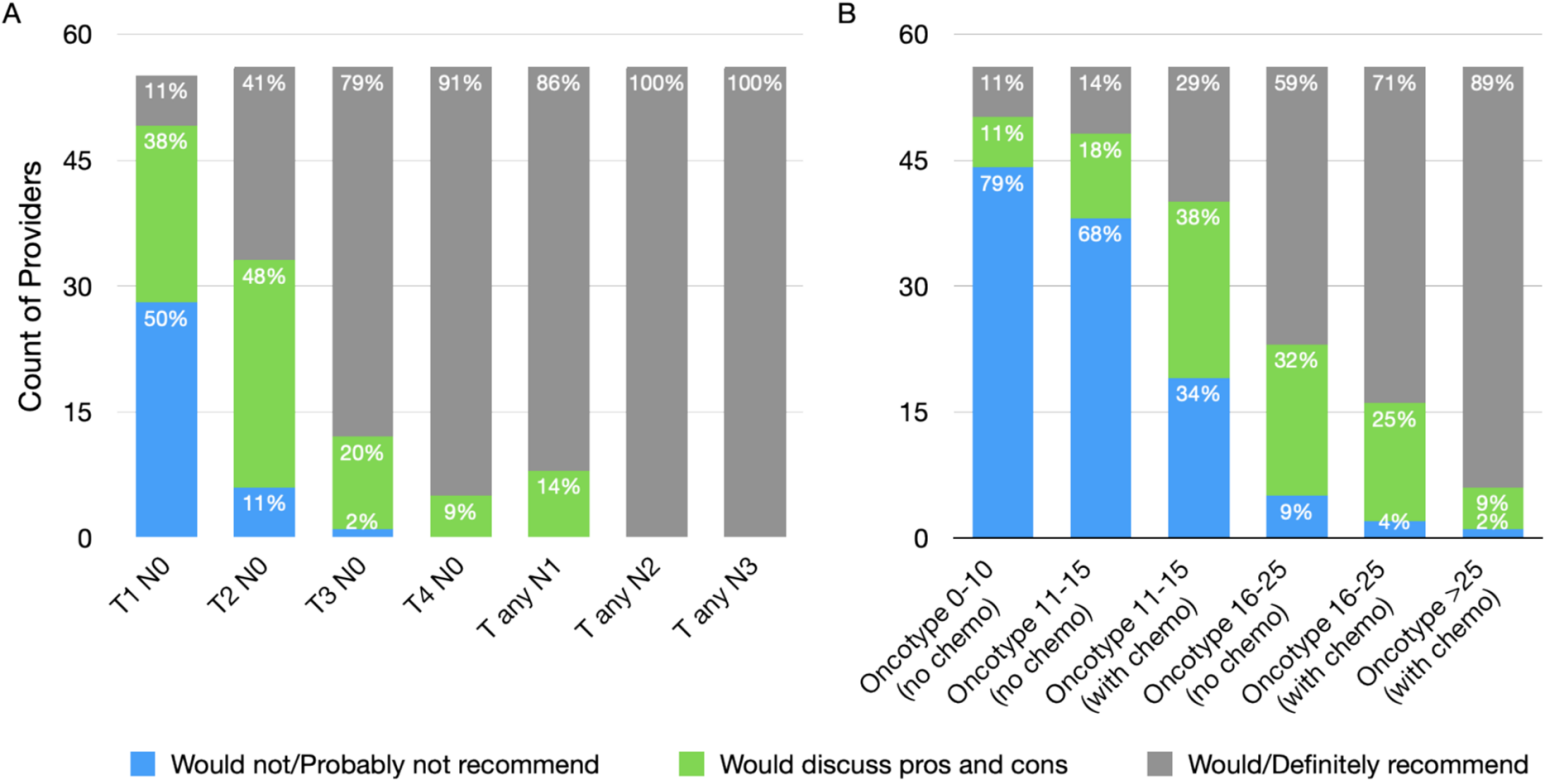
Responses to question asking how strongly oncologists would recommend OFS for a pre-/peri-menopausal woman with HR + early-stage operable breast cancer. **A** Responses by tumor stage. **B** Responses assuming patient has node-negative disease, by gene expression assay recurrence score Note: T1 N0 Missing = 1

Figure 1 shows trends of treatment recommendations considering both tumor staging and gene expression assay recurrence scores. As tumor size and nodal involvement increase, there was a shift toward stronger recommendations of OFS. A notable distinction emerged in treatment recommendations for node-negative patients with intermediate-low scores (11–15) depending on whether or not chemotherapy would be recommended. The majority (68%) of oncologists would not be likely to recommend OFS to these patients if they did not receive chemotherapy, but if chemotherapy was given then only 34% reported not being likely to recommend OFS with 38% reporting that they would now discuss the pros and cons of OFS. Chemotherapy was also frequently used as a surrogate for risk in our interviews. For example, one oncologist stated, “I think if they’re high enough risk to require chemotherapy, then I do strongly consider it [OFS].”

These findings were similar when we used clinical vignettes to summarize case details. The first clinical vignette was that of a 25-year-old woman with a screen-detected breast cancer status post-lumpectomy. Pathology showed a grade 2 invasive ductal carcinoma measuring 1.7 cm with 0/3 sentinel lymph nodes involved. The tumor was HR + HER2 negative (ER 100%, PR 100%, HER2 IHC 0), Ki67 10%, and oncotype score was 16. Most (62.5%) of our respondents would not have recommended chemotherapy to this patient. Of these, the recommendation for OFS was almost evenly split with 54% recommending OFS (+ AI/tamoxifen) and 46% recommending tamoxifen only. However, among the 21 oncologists who would have recommended chemotherapy for a similar patient, 95% would also recommend OFS.

The second clinical vignette was that of a 50-year-old pre-menopausal woman with screen-detected right breast cancer status post-lumpectomy which showed grade 3 invasive ductal carcinoma measuring 2.2 cm, 0/3 sentinel lymph nodes involved. The tumor was again HR + HER2 negative (ER 100%, PR 100%, HER2 IHC 0), Ki67 15%, and oncotype score was 21. In total, 61% of oncologists would not have recommended chemotherapy in this case, similar to the first. However, among the oncologists who would have opted against chemotherapy, 74% would have recommended OFS—a notable shift from the first vignette. Of the oncologists who would recommend chemotherapy, 86% would recommend OFS.

Practitioners with 10 or fewer years of oncology experience had a strong preference for recommending OFS as initial endocrine therapy regardless of chemotherapy exposure. All of these oncologists (100%) practitioners would have recommended OFS to patients in both vignettes if they had recommended chemotherapy and about 2/3rds would have recommended OFS to patients in both vignettes if chemotherapy was not recommended. In contrast, among oncologists with more than 10 years of oncology experience, approximately 60% favored tamoxifen alone when not recommending chemotherapy for the patient in the first vignette.

### Preferences regarding OFS type, timing, and schedule

**Fig. 2:**
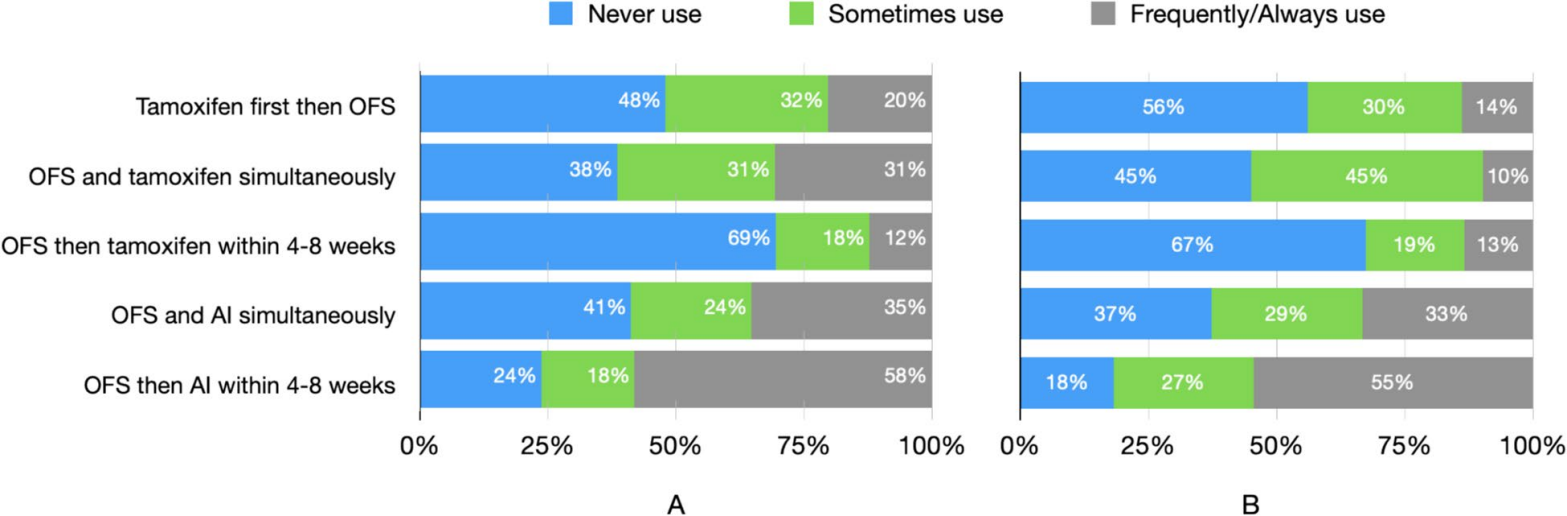
The rank of treatment plans that oncologists typically use for **A** pre-menopausal women with HR + early-stage breast cancer, when planning to use OFS and endocrine therapy after chemotherapy, and for **B** peri-menopausal women >  = 45, with HR + early-stage breast cancer, when planning to use OFS and endocrine therapy without chemotherapy. Notes: Missing responses in Panel A: tamoxifen first then OFS (*n* = 14), OFS and tamoxifen simultaneously (*n* = 4), OFS then tamoxifen within 4–8 weeks (*n* = 7), OFS and AI simultaneously (*n* = 5), and OFS then AI within 4–8 weeks (*n* = 1). Missing responses in Panel B: tamoxifen first then OFS (*n* = 6), OFS and tamoxifen simultaneously (*n* = 5), OFS then tamoxifen within 4–8 weeks (*n* = 4), OFS and AI simultaneously (*n* = 5), and OFS then AI within 4–8 weeks (*n* = 1)

There was a high level of consistency among practitioners regarding the use of OFS medications and dosing schedules for pre-/peri-menopausal women with early-stage, HR + breast cancer. The data showed that 94.6% of practitioners prefer either Leuprolide (50%) or Goserelin (44.6%), while other medications such as Triptorelin and Degarelix are used much less frequently. Monthly dosing is preferred (71.4%) to the 3-month schedule (28.6%). Regarding initiation of OFS and endocrine therapy, most oncologists will begin OFS first and then add AI (Fig. 2).

### Resources for OFS decision-making

Most practitioners determined their management plans based on established guidelines (NCCN/ASCO, 42.9%) or clinical trial data (SOFT/TEXT, 26.8%). Eighteen percent used prognostic calculators, such as CTS5, Predict, or RSClin. The majority of respondents (59%) reported that current NCCN guidelines regarding OFS use lack clarity and 70% report lack of clarity in current guidelines as a barrier to OFS use (Table 2).

**Table 2:** Barriers to OFS utilization

Barrier	Not a barrier N (%)	Less/somewhat important barrier N (%)	Very important barrier N (%)
Lack of clarity in professional guidelines^1^	16 (29%)	34 (61%)	5 (9%)
Lack of clinical knowledge about OFS use	28 (50%)	26 (46%)	2 (4%)
Lack of experience using OFS medications^1^	41 (73%)	12 (21%)	2 (4%)
Uncertainty about stopping OFS once it's started	16 (29%)	33 (59%)	7 (13%)
Concern about overtreatment	10 (18%)	37 (66%)	9 (16%)
Concerns about adherence to treatment plan	6 (11%)	42 (75%)	8 (14%)
Concerns about toxicities	3 (5%)	33 (59%)	20 (36%)
Concerns about burden to patient such as travel or time	5 (9%)	43 (77%)	8 (14%)
Concerns about cost/lack of insurance^1^	9 (16%)	37 (66%)	9 (16%)
Concerns raised by patient/Patient declining	3 (5%)	41 (73%)	12 (21%)

Note^1^: There was 1 missing data point, respectively, for these categories

### Determining menopausal status after chemotherapy and monitoring ovarian function

**Fig. 3:**
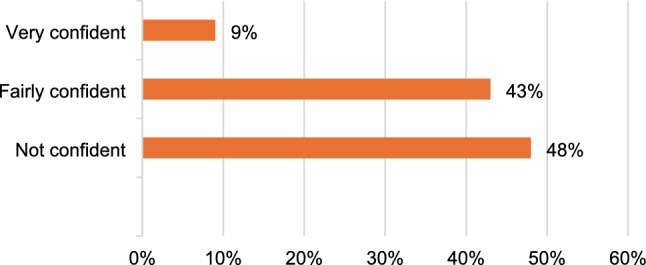
Confidence in confirming menopausal status post-chemotherapy

About half of our respondents (53%) used menopausal status at diagnosis to determine menopausal status after chemotherapy (Table 3). That is, if patients were pre-/peri-menopausal at diagnosis, they considered them to be pre-menopausal after chemotherapy even if they were amenorrheic. Thirty-eight percent would check serum estradiol and/or FSH and/or LH and base menopausal status on these lab values. However, only 9% of practitioners felt very confident confirming menopausal status in pre-menopausal women (at diagnosis) with amenorrhea post-chemotherapy (Fig. 3).

**Table 3:** Responses regarding determination of menopausal status after chemotherapy

	N (%)
I only use menopausal status at diagnosis. That is, if they were per-/peri-menopausal at diagnosis, I consider them to be pre-/peri-menopausal after chemotherapy even if they’re amenorrheic	29 (53%)
I check serum estradiol and/or FSH, and/or LH and base menopausal status on these lab values	21 (38%)
Other*	5 (9%)
If amenorrheic at the time of initiation of endocrine therapy, then I consider them post-menopausal	0

^*^Other assessment methods: I check estradiol, FSH, LH q6m × 1 year and consider them post-menopausal if labs are consistent over that time frame (*n* = 1); amenorrhea for 1 year, then check estradiol level (*n* = 1); serum levels at multiple time points to confirm (*n* = 1); use labs at diagnosis, but recheck in 1 year if menopause post-chemo suspected (*n* = 1); it depends (*n* = 1)

The approach to assessing ovarian suppression status after patients initiate OFS varied significantly, with 44.6% reassessing every 3–6 months, 21.4% never reassessing, 19.6% annually, 3.6% monthly, and 10.7% use other frequencies. Our data also demonstrated that most respondents indicated a need for greater specificity in guidelines regarding the initial assessment of menopausal status post-chemotherapy (79%), the assessment of recovery of ovarian function post-chemotherapy (81%), and assessment of ovarian function in peri-menopausal women on treatment with ET ± OFS (89%). This theme was also observed in our qualitative data, with one oncologist describing: “I don’t know if we have good guidelines on when to stop, when to test, how long they should be off their ovarian function suppression before we test hormones. So, I get confused.”

## Discussion

Our results suggest that practicing medical oncologists are likely to recommend OFS to pre-menopausal women at high risk for recurrence of HR + breast cancer. Larger tumor size, increased nodal burden, higher recurrence scores, and receipt of chemotherapy were all found to be consistently appreciated surrogates for high-risk disease with corresponding increases in the strength of recommendation for OFS within each risk category. These findings are consistent with current guidelines regarding risk factors for high-risk disease [3]. However, we also found that only 9% of practitioners felt confident in how to confirm menopausal status following chemotherapy.

The current NCCN guidelines state that “FSH and estradiol should be repeated serially to ensure menopausal status in patients with breast cancer with chemotherapy-induced amenorrhea.”[4] NCCN guidelines also recommend assessment ovarian function “prior to next dose of GnRH agonist especially in women younger than 45 years with frequency of testing individualized” and current ASCO guidelines alert clinicians about the risk of incomplete ovarian suppression with GnRHa and recommend evaluation of patients at risk for residual ovarian function [3]. While these guidelines appropriately leave room for individualized decision-making, our results support a need for more clarity regarding recommendations for assessment of ovarian function in the setting of chemotherapy-induced amenorrhea and for ovarian function monitoring for patients on OFS. Most medical oncologists in this study reported challenges in determining menopausal status post-chemotherapy. Only 9% of this sample reported confidence in determining menopausal status in the setting of amenorrhea post-chemotherapy and 79% endorsed a need for more specificity in guidelines regarding this topic. A recent survey study by Kelly and colleagues reported similar findings, with 55% of their respondents reporting challenges when interpreting hormonal profiles [7]. This is an important finding clinically, as the determination of menopausal status has significant implications for patient outcomes and incorrect assessment can lead to either undertreatment and worse breast cancer outcomes or overtreatment and associated symptoms, time toxicity, and financial burdens. Practices for monitoring ovarian function when patients are on OFS also varied widely in this sample. This remains an important avenue for future research as up to 27% of patients on OFS have inadequate ovarian suppression [8, 9]. The potential implications of incomplete ovarian suppression are likely most important for patients receiving OFS with an aromatase inhibitor as the biologic effectiveness of an aromatase inhibitor in a woman with preserved ovarian function is questionable.

Medical oncologists in this study report concerns about overtreating young women with HR + breast cancer. This is important to note as it may suggest negative attitudes toward OFS which could potentially impact strength of recommendation, and physicians’ recommendations have been positively correlated with the patient’s treatment preference [10, 11]. In another study, 92% of medical oncologists surveyed indicated that they would benefit from guidance regarding ovarian suppression [7]. Our findings lend support to a need for improved clarity in guidelines or professional resources such as shared decision-making guides could help to identify those at highest risk and most likely to benefit from OFS and therefore mitigate this concern as a potential barrier to OFS utilization. The ongoing OFSET phase III clinical trial will answer the question of whether the benefit of chemotherapy seen in certain subsets of pre-menopausal women with HR + HER2-negative breast cancer is due to ovarian ablative effects of chemotherapy [5]. If this proves to be the case and chemotherapy can be omitted for this subset of patients, then ideal use of and adherence to OFS and endocrine therapy will become even more vital to ensuring optimal patient outcomes.

Treatment toxicity was another frequently reported barrier to OFS use. Clinical trials have demonstrated significant increases in toxicity with the addition of OFS to ET [12, 13]. Toxicities include health related quality of life, menopausal symptoms, vaginal dryness, and sexual function and many of these side effects persist for the duration of treatment. These treatment related symptoms resulted in approximately 20% of women in the SOFT trial discontinuing OFS early [13].

Patient-level factors were also identified as potential barriers to OFS use with most respondents reporting their concerns about adherence, general patient concerns about OFS, and patients outright declining OFS as potential barriers to OFS use. Little is known about patient-level decision-making regarding OFS. Future work is needed to explore patient-level factors contributing to OFS utilization.

## Strengths and limitations

This study has several limitations including small sample size and low response rates which may limit generalizability of the findings. However, this is a foundational exploratory study and the first study to explore oncologist factors regarding OFS use in patients with breast cancer. Furthermore, the strength of the data collected is enhanced by the survey’s development, which was guided by stakeholder interviews.

## Conclusion

Medical oncologists in our sample generally agreed with recommendations for use of OFS among women at high risk for recurrence of HR + breast cancer. We identified wide variations in monitoring ovarian function on OFS, and oncologists indicated a need for clearer guidance on determining menopausal status to establish OFS candidacy and OFS monitoring.

## Supplementary Information

Below is the link to the electronic supplementary material.

Supplementary file1 (PDF 199 kb)

## Data Availability

The datasets generated during the current study are not openly available due to reasons of sensitivity but are available from the corresponding author on reasonable request.
