# Supplementary material for: A mixed-methods study characterizing experiences of medical oncologists’ use of gonadotropin-releasing hormone agonists for treatment of breast cancer
Source: Breast Cancer Res Treat. 2025 Jun 3;212(3):449–55. doi: 10.1007/s10549-025-07734-2 (PMC12209034; doi:10.1007/s10549-025-07734-2)
Supplement: Supplementary file 1 — Supplementary file1 (PDF 199 kb) [file 10549_2025_7734_MOESM1_ESM.pdf]

MCC 22572
Aim 2: Provider survey

The aim of this survey is to learn more about medical oncologists use ovarian function suppression for pre-menopausal women with hormone receptor positive breast cancer. We are also trying to understand how OFS is monitoring in real-world practice. If you decide to take part in our study, you would be providing information that may help to improve the use of OFS among young women with breast cancer.

[Screening question] Would you estimate that at least 15% of your patients have breast cancer?

□ Yes – If yes, move onto survey

□ No – If no, then they should see a message. “Thank you for your time, but unfortunately, you do not qualify for participation in this study.”

DEMOGRAPHIC QUESTIONS

**What is your Gender?**<br>□ Male<br>□ Female<br>□ Other (please specify)__________
**What is your Race?**<br>□ White<br>□ Black or African American;<br>□ Asian;<br>□ American Indian or Alaskan Native;<br>□ Native Hawaiian or Pacific Islander;<br>□ Other (please specify) _______________________
**What is your Ethnicity?**<br>□ Hispanic or Latino;<br>□ Not Hispanic or Latino;<br>□ Caribbean descent;<br>□ Middle Eastern North African descent
**How many years have you been in practice (after completion of medical training)?**<br>□ 0-5 years,<br>□ 5-10 years,<br>□ >10 years
**What is your practice type?**<br>□ Academic Medical Center: I work in an academic medical center associated with a university or research institution.<br>□ Community Hospital: I am part of a medical practice located in a community hospital setting.<br>□ Private Practice: I operate my own private practice independently or in a group setting.<br>□ Government or Public Health Institution: I am associated with a government or public health institution that provides oncology services.

CLINICAL VIGNETTES

Questions below indicated as “SCENARIO” present patient scenarios and ask how you would manage a patient with these results.

MCC 22572
Aim 2: Provider survey

SCENARIO A: A 25-year-old with a recent diagnosis of a right breast cancer found on screening mammogram had a lumpectomy which showed grade 2 invasive ductal carcinoma measuring 1.7 cm, 0/3 sentinel lymph nodes involved, pT1c N0. ER 100%, PR 100%, HER2 IHC 0. Ki67 10%. Oncotype score was 16.

3. Would you recommend chemotherapy?
   - Yes
   - No
4. A. [If "Yes" to number 3] What would you recommend for initial endocrine therapy assuming she does not have chemotherapy induced ovarian failure?
   - Tamoxifen only
   - Tamoxifen + Ovarian Function Suppression
   - Aromatase inhibitor + Ovarian Function Suppression

   4B. [If "No" to number 3] What would you recommend for initial endocrine therapy?

SCENARIO B: A 50 year old with a recent diagnosis of a right breast cancer found on screening mammogram had a lumpectomy which showed grade 3 invasive ductal carcinoma measuring 2.2 cm, 0/3 sentinel lymph nodes involved, pT2 N0. ER 100%, PR 100%, HER2 IHC 0. Ki67 15%. Oncotype score was 21.

5. Would you recommend chemotherapy?
   Yes
   No

6. A. [If "Yes" to number 5] What would you recommend for initial endocrine therapy assuming she does not have chemotherapy induced ovarian failure?
   a. Tamoxifen only
   b. Tamoxifen + Ovarian Function Suppression
   c. Aromatase inhibitor + Ovarian Function Suppression

   6B. What would you recommend for initial endocrine therapy?
   a. Tamoxifen only
   b. Tamoxifen + Ovarian Function Suppression
   c. Aromatase inhibitor + Ovarian Function Suppression

7. How would you typically determine your management plans for the patients in Scenarios A and B?
   - NCCN/ASCO guidelines
   - SOFT/TEXT trial data
   - Calculators such as CTS5, Predict, or RSClin
   - Guideline from my own institution or healthcare system
   - Expert opinion as obtained from conference or CME attendance
   - Other (please specify):

OFS DECISION MAKING

8. For a pre-/peri-menopausal woman with early stage, hormone receptor positive (HR+) breast cancer, which ovarian function suppression medication do you use most often?
    - Goserelin
    - Luprolide
    - Triptorelin
    - Degarelix
    - Other, please specify

9. When you use OFS, which dosing schedule do you use more often?
    - Monthly
    - Every 3 months

How strongly would you recommend OFS for a pre-/peri-menopausal woman with HR+ early stage operable breast cancer with the following characteristics:

Answer choices: 1) Would not recommend 2) Probably would not recommend 3) Would discuss pros and cons 4) Probably would recommend 5) Would definitely recommend

10. T1, N0
11. T2, N0
12. T3, N0
13. T4, N0
    [NEXT Page]

14. T any, N1
15. T any, N2
16. T any, N3

17. How strongly would you recommend OFS for a pre-/peri-menopausal woman with a node negative, HR+ early stage breast cancer with the following characteristics:
    Answer choices: 1) Would not recommend 2) Probably would not recommend 3) Would discuss pros and cons 4) Probably would recommend 5) Would definitely recommend
    - Oncotype score 0-10, did not receive chemotherapy
    - Oncotype score 11-15, did not receive chemotherapy
    - Oncotype score 11-15, received chemotherapy
    - Oncotype score 11-15, received chemotherapy
    - Oncotype score 16-25, did not receive chemotherapy
    - Oncotype score 16-25, received chemotherapy
    - Oncotype score >25, received chemotherapy

18. For premenopausal women with HR+ early stage breast cancer, when planning to use OFS and endocrine therapy after chemotherapy, please rank the treatment plan you typically use:
    1) Never use 2) Sometimes use 3) frequently use 4) Always use

    - I start tamoxifen. If menses return within 6-12 months, I start OFS
    - I start tamoxifen. If menses do not return within 6-12 months, I check estradiol levels and start OFS if estradiol level confirms premenopausal status
    - I start OFS and tamoxifen at the same time
    - I start OFS, then Tamoxifen within 4-8 weeks
    - I start OFS and Aromatase Inhibitor at the same time
    - I start OFS, then Aromatase Inhibitor within 4-8 weeks
    - Other, please specify

19. For peri-menopausal women >=45, with HR+ early stage breast cancer, when planning to use OFS and endocrine therapy without chemotherapy, please rank the treatment plan you typically use:
    1) Never use 2) Sometimes use 3) frequently use 4) Always use
    - I start tamoxifen first, then add OFS.
    - I start OFS and tamoxifen at the same time
    - I start OFS, then Tamoxifen within 4-8 weeks
    - I start OFS and Aromatase Inhibitor at the same time
    - I start OFS, then Aromatase Inhibitor within 4-8 weeks

20. In what circumstances would you typically discontinue OFS in a patient who is tolerating treatment well?
    - After 7-10 years of OFS
    - After 5 years of OFS
    - In a woman who was perimenopausal at diagnosis, I stop OFS if no menses for >= 12 months
    - In a woman who was perimenopausal at diagnosis, I stop OFS after 2 years
    - Other, please specify

21. Current NCCN guidelines recommend ovarian suppression or ablation for premenopausal woman at higher risk for recurrence (young age, high-grade tumor, lymph node involvement). Do you think current guidelines regarding OFS use offer enough clarity with regards to when to use these medications?
    a. Yes
    b. No
    c. Unsure

To what extent do you view each of the following as potential barriers to your routine use of OFS for high-risk pre-menopausal patients?

	Not a barrier	Less important barrier	Somewhat important barrier	Very important barrier

22. Lack of clarity in professional guidelines				
23. Lack of clinical knowledge about OFS use				
24. Lack of experience using OFS medications				
25. Uncertainty about stopping OFS once it's started				
26. Concern about overtreatment				
27. Concerns about adherence to treatment plan				
28. Concerns about toxicities				
29. Concerns about burden to patient, travel, time				
30. Cost/lack of insurance				
31. Concerns raised by patient/Patient declining				

How strongly would you recommend pre-menopausal patients with HR+ early stage breast cancer consider bilateral salpingo-oophorectomy?

Clinical features	(1) Would not recommend	Consider recommending (2)	(3) Strongly recommend
32. If they are very young (<35 years) at diagnosis			
33. If they are peri-menopausal or >45 years at diagnosis			
34. If they do not tolerate OFS medication or tamoxifen			
35. If patient prefers surgery to avoid OFS medication			
36. If patient has a germline mutation that increases risk of ovarian cancer			
37. If patient has a family history of ovarian cancer			

ASSESSING MENOPAUSAL STATUS AND OVARIAN FUNCTION

38. For patients who were premenopausal at diagnosis, how do you confirm menopausal status after chemotherapy?
    - I only use menopausal status at diagnosis. That is, if they were pre-/peri-menopausal at diagnosis, I consider them to be pre-/peri-menopausal after chemotherapy even if they're amenorrheic
    - If amenorrheic at the time of initiation of endocrine therapy, then I consider them post-menopausal
    - I check serum estradiol and/or FSH, and/or LH and base menopausal status on these lab values
    - Other [option to type in answer]

39. How confident do you feel confirming menopausal status in pre-menopausal women (at diagnosis) with amenorrhea post-chemotherapy?
    a. Not confident
    b. Fairly confident
    c. Very confident

40. How often do you reassess ovarian suppression status after a pre-menopausal woman has initiated ovarian function suppression for treatment of HR+ early breast cancer?
    a. Monthly
    b. Every 3-6 months
    c. Annually
    d. Never
    e. Other, please specify

41. Would you benefit from more specificity in guidelines regarding the initial assessment of menopausal status post-chemotherapy?
    a. Yes
    b. No
    c. Unsure

42. Would you benefit from more specificity in guidelines regarding assessment of recovery of ovarian function post-chemotherapy?
    a. Yes
    b. No
    c. Unsure

43. Would you benefit from more specificity in guidelines regarding assessment of ovarian function in peri-menopausal women on treatment with ET +/- OFS. That is, whether or not a peri-menopausal woman at treatment initiation has undergone natural menopause during treatment?
    a. Yes
    b. No
    c. Unsure

SYMPTOM MANAGEMENT

44. How comfortable do you feel managing the following side effects of endocrine therapy? That is, would you prefer to manage these, or refer if possible?

**Symptoms**	1<br>Very uncomfortable	2<br>Uncomfortable	3<br>Neutral	4<br>Comfortable	5<br>Very comfortable
Vasomotor symptoms					
Vaginal dryness /Dyspareunia					
Low libido					
Anxiety					
Depression					
Osteopenia/ osteoporosis					
Arthralgias					

45. On a scale from 1 to 5, How confident are you in discussing and recommending the following treatments for vaginal dryness/dyspareunia secondary to endocrine therapy.

Treatments	1<br>Not confident	2<br>Fairly confident	3<br>Very confident
Vaginal moisturizers			
Vaginal hyaluronic acid			
Vaginal estrogen			
